# [μ-1,6-Bis(diphenyl­arsan­yl)hexa­ne]bis­[chloridogold(I)]

**DOI:** 10.1107/S1600536811008646

**Published:** 2011-03-12

**Authors:** Omar bin Shawkataly, Abu Tariq, Imthyaz Ahmad Khan, Chin Sing Yeap, Hoong-Kun Fun

**Affiliations:** a Chemical Sciences Programme, School of Distance Education, Universiti Sains Malaysia, 11800 USM, Penang, Malaysia; b X-ray Crystallography Unit, School of Physics, Universiti Sains Malaysia, 11800 USM, Penang, Malaysia

## Abstract

In the title compound, [Au_2_Cl_2_(C_30_H_32_As_2_)], each Au atom is coordinated by As and Cl atoms in an approximately linear geometry. In the crystal, mol­ecules are linked into two-dimensional networks parallel to the *ac* plane *via* inter­molecular C—H⋯Cl inter­actions. One of the phenyl rings is disordered over two positions, with site occupancies of 0.518 (8) and 0.482 (8).

## Related literature

For general background and applications of diphenyl­arsino derivatives, see: Hill *et al.* (1983 ▶). For general background and applications of gold(I) complexes, see: Parish & Cottrill (1987 ▶); Tiekink (2002 ▶). For the synthesis of (CH_3_)_2_SAuCl, see: Francis (1901 ▶). For the synthesis of 1,6-bis­(diphenyl­arsino)hexane, see: Shawkataly *et al.* (2009 ▶). For a closely related structure, see: Shawkataly *et al.* (2010 ▶). For the stability of the temperature controller used in the data collection, see: Cosier & Glazer (1986 ▶). For a description of the Cambridge Structural Database, see: Allen (2002 ▶).
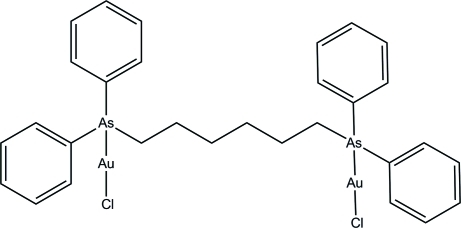


## Experimental

### 

#### Crystal data

[Au_2_Cl_2_(C_30_H_32_As_2_)]
*M* *_r_* = 1007.23
Triclinic, 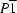

*a* = 9.4881 (3) Å
*b* = 11.0350 (4) Å
*c* = 15.5254 (5) Å
α = 69.723 (1)°
β = 83.959 (1)°
γ = 79.814 (1)°
*V* = 1499.06 (9) Å^3^
*Z* = 2
Mo *K*α radiation
μ = 12.16 mm^−1^
*T* = 100 K
0.37 × 0.22 × 0.09 mm

#### Data collection

Bruker SMART APEXII CCD area-detector diffractometer
Absorption correction: multi-scan (*SADABS*; Bruker, 2009 ▶) *T*_min_ = 0.094, *T*_max_ = 0.407
23816 measured reflections
7451 independent reflections
6817 reflections with *I* > 2σ(*I*)
*R*_int_ = 0.027

#### Refinement

*R*[*F* ^2^ > 2σ(*F* ^2^)] = 0.031
*wR*(*F* ^2^) = 0.088
*S* = 1.09
7451 reflections
371 parameters
H-atom parameters constrained
Δρ_max_ = 2.52 e Å^−3^
Δρ_min_ = −3.00 e Å^−3^

### 

Data collection: *APEX2* (Bruker, 2009 ▶); cell refinement: *SAINT* (Bruker, 2009 ▶); data reduction: *SAINT*; program(s) used to solve structure: *SHELXTL* (Sheldrick, 2008 ▶); program(s) used to refine structure: *SHELXTL*; molecular graphics: *SHELXTL*; software used to prepare material for publication: *SHELXTL* and *PLATON* (Spek, 2009) ▶.

## Supplementary Material

Crystal structure: contains datablocks I, global. DOI: 10.1107/S1600536811008646/is2685sup1.cif

Structure factors: contains datablocks I. DOI: 10.1107/S1600536811008646/is2685Isup2.hkl

Additional supplementary materials:  crystallographic information; 3D view; checkCIF report

## Figures and Tables

**Table:** 

Au1—Cl1	2.3043 (10)
Au1—As1	2.3411 (4)
Au2—Cl2	2.3005 (10)
Au2—As2	2.3398 (5)

**Table:** 

Cl1—Au1—As1	174.77 (3)
Cl2—Au2—As2	175.14 (3)

**Table 2:** Hydrogen-bond geometry (Å, °)

*D*—H⋯*A*	*D*—H	H⋯*A*	*D*⋯*A*	*D*—H⋯*A*
C17—H17*B*⋯Cl2^i^	0.97	2.79	3.754 (5)	172
C18—H18*A*⋯Cl1^ii^	0.97	2.80	3.701 (5)	155

Symmetry codes: (i) 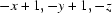; (ii) 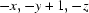.

**Fig. 1.**
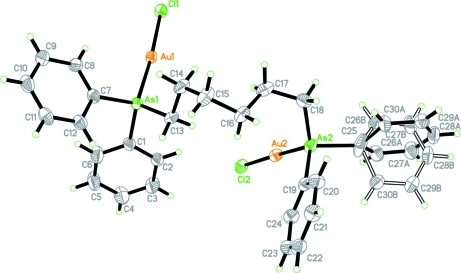
The molecular structure of title compound, with 30% probability ellipsoid for non-H atoms and the atom-numbering scheme.

**Fig. 2.**
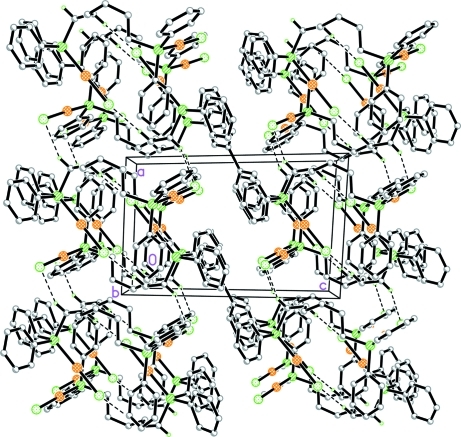
The crystal packing of the title compound, viewed along the b axis. Intermolecular interactions are shown as dashed lines.

## supplementary crystallographic information

### Comment

Gold and gold complexes have been used for medicinal purposes over a long period of time (Parish & Cottrill, 1987; Tiekink, 2002). 1,6-Bis(diphenylarsino)hexane has been used for *trans* chelation in transition metal complexes (Hill *et al.*, 1983). A search of the November 2010 release of the Cambridge Structural Database (Allen, 2002) revealed no such gold (I) complexes containing the above ligand has been reported. Herein, we report the crystal structure of the title complex (Ph)_2_As(CH_2_)_6_As(Ph)_2_Au_2_Cl_2_ (Fig. 1).

The As1—Au1—Cl1 is almost linear with an angle of 174.77 (3)° and As2—Au2—Cl2 with an angle of 175.14 (3)°. The four substituted phenyl rings on both arsines C1—C6/C7—C12, C19—C24/C25,C26B—C30B and C19—C24/C25,C26A—C30A are inclined to one another, with dihedral angles of 69.1 (4), 85.9 (4) and 73.4 (4)°, respectively.

In the crystal packing, (Fig. 2), the molecules are linked into two-dimensional networks parallel to the (010) plane *via* intermolecular C17—H17B···Cl2 and C18—H18A···Cl1 interactions (Table 2).

### Experimental

(Ph)_2_As(CH_2_)_6_As(Ph)_2_AuCl was prepared by mixing equimolar quantities of Me_2_SAuCl, obtained as per conventional method (Francis, 1901) and (Ph)_2_As(CH_2_)_6_As(Ph)_2_, synthesized according to related literature (Shawkataly *et al.*, 2009) in CH_2_Cl_2_ held at room temperature. The solution was stirred for 2 h, and white crystalline solid was recovered after the removal of solvent under vacuum. The colourless plate-like crystals were obtained in 90% yield by solvent/solvent diffusion of dichloromethane/methanol at 10 °C after 2 days (m.p. 203 °C).

### Refinement

All atoms are positioned geometrically (C—H = 0.93 or 0.97 Å) and refined using a riding model, with *U*_iso_(H) = 1.2*U*_eq_(C). One out of four phenyl rings is disordered over two positions with refined site-occupancies of 0.518 (8):0.482 (8). The maximum and minimum residual electron density peaks were located 0.84 and 0.86 Å, respectively, from atom Au1.

### Crystal data

**Table:**

[Au_2_Cl_2_(C_30_H_32_As_2_)]	*Z* = 2
*M**_r_* = 1007.23	*F*(000) = 940
Triclinic, *P*1	*D*_x_ = 2.231 Mg m^−^^3^
Hall symbol: -P 1	Mo *K*α radiation, λ = 0.71073 Å
*a* = 9.4881 (3) Å	Cell parameters from 9948 reflections
*b* = 11.0350 (4) Å	θ = 3.2–35.1°
*c* = 15.5254 (5) Å	µ = 12.16 mm^−^^1^
α = 69.723 (1)°	*T* = 100 K
β = 83.959 (1)°	Plate, colourless
γ = 79.814 (1)°	0.37 × 0.22 × 0.09 mm
*V* = 1499.06 (9) Å^3^	

### Data collection

**Table:**

Bruker SMART APEXII CCD area-detector diffractometer	7451 independent reflections
Radiation source: fine-focus sealed tube	6817 reflections with *I* > 2σ(*I*)
graphite	*R*_int_ = 0.027
φ and ω scans	θ_max_ = 28.5°, θ_min_ = 2.5°
Absorption correction: multi-scan (*SADABS*; Bruker, 2009)	*h* = −12→12
*T*_min_ = 0.094, *T*_max_ = 0.407	*k* = −14→14
23816 measured reflections	*l* = −20→20

### Refinement

**Table:**

Refinement on *F*^2^	Primary atom site location: structure-invariant direct methods
Least-squares matrix: full	Secondary atom site location: difference Fourier map
*R*[*F*^2^ > 2σ(*F*^2^)] = 0.031	Hydrogen site location: inferred from neighbouring sites
*wR*(*F*^2^) = 0.088	H-atom parameters constrained
*S* = 1.09	*w* = 1/[σ^2^(*F*_o_^2^) + (0.0596*P*)^2^ + 0.921*P*] where *P* = (*F*_o_^2^ + 2*F*_c_^2^)/3
7451 reflections	(Δ/σ)_max_ = 0.001
371 parameters	Δρ_max_ = 2.52 e Å^−^^3^
0 restraints	Δρ_min_ = −3.00 e Å^−^^3^

### Special details

**Table:**

Experimental. The crystal was placed in the cold stream of an Oxford Cyrosystems Cobra open-flow nitrogen cryostat (Cosier & Glazer, 1986) operating at 100.0 (1) K.
Geometry. All e.s.d.'s (except the e.s.d. in the dihedral angle between two l.s. planes) are estimated using the full covariance matrix. The cell e.s.d.'s are taken into account individually in the estimation of e.s.d.'s in distances, angles and torsion angles; correlations between e.s.d.'s in cell parameters are only used when they are defined by crystal symmetry. An approximate (isotropic) treatment of cell e.s.d.'s is used for estimating e.s.d.'s involving l.s. planes.
Refinement. Refinement of *F*^2^ against ALL reflections. The weighted *R*-factor *wR* and goodness of fit *S* are based on *F*^2^, conventional *R*-factors *R* are based on *F*, with *F* set to zero for negative *F*^2^. The threshold expression of *F*^2^ > σ(*F*^2^) is used only for calculating *R*-factors(gt) *etc*. and is not relevant to the choice of reflections for refinement. *R*-factors based on *F*^2^ are statistically about twice as large as those based on *F*, and *R*- factors based on ALL data will be even larger.

### Fractional atomic coordinates and isotropic or equivalent isotropic displacement parameters (Å^2^)

**Table:**

	*x*	*y*	*z*	*U*_iso_*/*U*_eq_	Occ. (<1)
Au1	0.293305 (16)	0.506581 (14)	−0.244387 (10)	0.02413 (6)	
Au2	0.485482 (16)	0.400959 (15)	0.158348 (11)	0.02685 (6)	
As1	0.35104 (4)	0.28305 (4)	−0.16352 (3)	0.02363 (9)	
As2	0.29128 (5)	0.36296 (4)	0.26530 (3)	0.02696 (10)	
Cl1	0.22329 (12)	0.72149 (10)	−0.33185 (7)	0.0304 (2)	
Cl2	0.67793 (11)	0.42021 (11)	0.05371 (7)	0.0313 (2)	
C1	0.5539 (4)	0.2189 (4)	−0.1582 (3)	0.0287 (8)	
C2	0.6304 (4)	0.2508 (4)	−0.0994 (3)	0.0295 (8)	
H2A	0.5832	0.2955	−0.0612	0.035*	
C3	0.7788 (5)	0.2148 (6)	−0.0986 (4)	0.0404 (11)	
H3A	0.8309	0.2350	−0.0592	0.048*	
C4	0.8488 (5)	0.1492 (7)	−0.1559 (5)	0.0505 (15)	
H4A	0.9481	0.1272	−0.1560	0.061*	
C5	0.7712 (6)	0.1161 (7)	−0.2133 (5)	0.0550 (16)	
H5A	0.8184	0.0697	−0.2505	0.066*	
C6	0.6239 (5)	0.1517 (6)	−0.2155 (4)	0.0438 (12)	
H6A	0.5722	0.1310	−0.2548	0.053*	
C7	0.2716 (4)	0.1765 (4)	−0.2167 (3)	0.0273 (8)	
C8	0.1984 (5)	0.2365 (5)	−0.2958 (3)	0.0323 (9)	
H8A	0.1872	0.3270	−0.3228	0.039*	
C9	0.1405 (5)	0.1616 (5)	−0.3361 (4)	0.0399 (11)	
H9A	0.0909	0.2020	−0.3897	0.048*	
C10	0.1578 (5)	0.0266 (5)	−0.2953 (4)	0.0375 (11)	
H10A	0.1202	−0.0234	−0.3221	0.045*	
C11	0.2301 (5)	−0.0338 (5)	−0.2156 (4)	0.0347 (10)	
H11A	0.2404	−0.1242	−0.1886	0.042*	
C12	0.2882 (4)	0.0404 (4)	−0.1750 (3)	0.0301 (8)	
H12A	0.3372	−0.0001	−0.1211	0.036*	
C13	0.2832 (4)	0.2282 (4)	−0.0364 (3)	0.0278 (8)	
H13A	0.3047	0.1339	−0.0098	0.033*	
H13B	0.3323	0.2661	−0.0025	0.033*	
C14	0.1221 (5)	0.2710 (5)	−0.0280 (3)	0.0342 (10)	
H14A	0.0762	0.2492	−0.0725	0.041*	
H14B	0.1037	0.3652	−0.0436	0.041*	
C15	0.0537 (5)	0.2088 (6)	0.0675 (3)	0.0415 (12)	
H15A	−0.0489	0.2383	0.0655	0.050*	
H15B	0.0685	0.1148	0.0816	0.050*	
C16	0.1099 (5)	0.2384 (5)	0.1452 (3)	0.0336 (9)	
H16A	0.0632	0.1921	0.2030	0.040*	
H16B	0.2120	0.2071	0.1490	0.040*	
C17	0.0834 (5)	0.3838 (5)	0.1310 (3)	0.0355 (10)	
H17A	−0.0134	0.4190	0.1109	0.043*	
H17B	0.1488	0.4266	0.0820	0.043*	
C18	0.1019 (5)	0.4173 (5)	0.2160 (3)	0.0331 (9)	
H18A	0.0334	0.3775	0.2638	0.040*	
H18B	0.0781	0.5112	0.2012	0.040*	
C19	0.3067 (5)	0.1805 (4)	0.3382 (3)	0.0279 (8)	
C20	0.1897 (5)	0.1239 (5)	0.3893 (3)	0.0334 (9)	
H20A	0.0996	0.1741	0.3874	0.040*	
C21	0.2088 (5)	−0.0067 (5)	0.4425 (3)	0.0359 (10)	
H21A	0.1307	−0.0449	0.4749	0.043*	
C22	0.3447 (5)	−0.0821 (5)	0.4480 (3)	0.0341 (9)	
H22A	0.3571	−0.1695	0.4851	0.041*	
C23	0.4612 (5)	−0.0265 (5)	0.3983 (3)	0.0333 (9)	
H23A	0.5516	−0.0765	0.4019	0.040*	
C24	0.4421 (5)	0.1041 (4)	0.3432 (3)	0.0316 (9)	
H24A	0.5199	0.1411	0.3092	0.038*	
C25	0.2720 (8)	0.4463 (5)	0.3575 (3)	0.0541 (17)	
C26A	0.3553 (10)	0.5057 (11)	0.3781 (8)	0.037 (2)	0.482 (8)
H26A	0.4447	0.5076	0.3471	0.044*	0.482 (8)
C27A	0.3295 (14)	0.5701 (13)	0.4428 (11)	0.043 (3)	0.482 (8)
H27A	0.4024	0.6061	0.4561	0.052*	0.482 (8)
C28A	0.1990 (16)	0.5802 (10)	0.4860 (7)	0.039 (3)	0.482 (8)
H28A	0.1837	0.6230	0.5290	0.047*	0.482 (8)
C29A	0.0882 (11)	0.5285 (10)	0.4680 (7)	0.036 (2)	0.482 (8)
H29A	−0.0009	0.5355	0.4988	0.043*	0.482 (8)
C30A	0.1120 (10)	0.4651 (10)	0.4023 (7)	0.033 (2)	0.482 (8)
H30A	0.0377	0.4348	0.3852	0.039*	0.482 (8)
C26B	0.2438 (11)	0.5670 (9)	0.3417 (7)	0.036 (2)	0.518 (8)
H26B	0.2253	0.6210	0.2817	0.043*	0.518 (8)
C27B	0.2385 (13)	0.6253 (10)	0.4094 (7)	0.042 (2)	0.518 (8)
H27B	0.1975	0.7120	0.3987	0.050*	0.518 (8)
C28B	0.2962 (14)	0.5497 (11)	0.4922 (9)	0.036 (2)	0.518 (8)
H28B	0.2954	0.5868	0.5377	0.044*	0.518 (8)
C29B	0.3554 (10)	0.4201 (10)	0.5098 (6)	0.037 (2)	0.518 (8)
H29B	0.3949	0.3715	0.5661	0.044*	0.518 (8)
C30B	0.3556 (10)	0.3630 (10)	0.4433 (6)	0.034 (2)	0.518 (8)
H30B	0.4034	0.2793	0.4500	0.041*	0.518 (8)

### Atomic displacement parameters (Å^2^)

**Table:**

	*U*^11^	*U*^22^	*U*^33^	*U*^12^	*U*^13^	*U*^23^
Au1	0.02936 (9)	0.02235 (9)	0.02283 (9)	−0.00656 (6)	−0.00161 (6)	−0.00885 (7)
Au2	0.02824 (9)	0.02498 (10)	0.02712 (10)	−0.00824 (6)	−0.00585 (6)	−0.00511 (7)
As1	0.02404 (18)	0.02315 (19)	0.0253 (2)	−0.00406 (14)	−0.00259 (14)	−0.00946 (16)
As2	0.0350 (2)	0.0243 (2)	0.0226 (2)	−0.00871 (16)	−0.00427 (16)	−0.00620 (16)
Cl1	0.0411 (5)	0.0226 (4)	0.0278 (5)	−0.0079 (4)	−0.0033 (4)	−0.0069 (4)
Cl2	0.0305 (5)	0.0361 (5)	0.0298 (5)	−0.0092 (4)	−0.0030 (4)	−0.0116 (4)
C1	0.0263 (18)	0.029 (2)	0.034 (2)	−0.0034 (15)	−0.0032 (16)	−0.0150 (18)
C2	0.0275 (19)	0.032 (2)	0.032 (2)	−0.0032 (16)	−0.0029 (16)	−0.0142 (18)
C3	0.029 (2)	0.053 (3)	0.050 (3)	−0.011 (2)	−0.0064 (19)	−0.026 (3)
C4	0.024 (2)	0.069 (4)	0.075 (4)	−0.004 (2)	−0.006 (2)	−0.046 (3)
C5	0.035 (2)	0.080 (4)	0.072 (4)	0.003 (3)	−0.004 (2)	−0.058 (4)
C6	0.033 (2)	0.061 (3)	0.053 (3)	−0.007 (2)	−0.002 (2)	−0.039 (3)
C7	0.0267 (18)	0.0243 (19)	0.035 (2)	−0.0064 (15)	−0.0007 (15)	−0.0135 (17)
C8	0.033 (2)	0.027 (2)	0.040 (2)	−0.0030 (16)	−0.0113 (18)	−0.0135 (19)
C9	0.040 (2)	0.040 (3)	0.048 (3)	−0.002 (2)	−0.013 (2)	−0.024 (2)
C10	0.030 (2)	0.039 (3)	0.055 (3)	−0.0072 (18)	0.0001 (19)	−0.030 (2)
C11	0.036 (2)	0.026 (2)	0.046 (3)	−0.0090 (17)	0.0064 (19)	−0.016 (2)
C12	0.0281 (19)	0.028 (2)	0.036 (2)	−0.0052 (16)	0.0008 (16)	−0.0132 (18)
C13	0.0281 (19)	0.028 (2)	0.024 (2)	−0.0030 (15)	−0.0063 (15)	−0.0047 (16)
C14	0.0244 (19)	0.053 (3)	0.024 (2)	−0.0065 (18)	−0.0038 (15)	−0.010 (2)
C15	0.036 (2)	0.065 (3)	0.027 (2)	−0.022 (2)	−0.0001 (18)	−0.013 (2)
C16	0.037 (2)	0.039 (2)	0.024 (2)	−0.0124 (19)	−0.0035 (16)	−0.0068 (18)
C17	0.030 (2)	0.043 (3)	0.029 (2)	−0.0099 (18)	−0.0066 (17)	−0.002 (2)
C18	0.031 (2)	0.031 (2)	0.032 (2)	−0.0022 (16)	0.0019 (17)	−0.0061 (18)
C19	0.037 (2)	0.025 (2)	0.0221 (19)	−0.0090 (16)	−0.0050 (16)	−0.0045 (16)
C20	0.031 (2)	0.031 (2)	0.035 (2)	−0.0039 (17)	−0.0010 (17)	−0.0074 (19)
C21	0.038 (2)	0.034 (2)	0.033 (2)	−0.0120 (19)	0.0027 (18)	−0.0069 (19)
C22	0.046 (3)	0.027 (2)	0.026 (2)	−0.0081 (18)	−0.0031 (18)	−0.0048 (18)
C23	0.036 (2)	0.030 (2)	0.033 (2)	−0.0034 (17)	−0.0032 (17)	−0.0093 (19)
C24	0.035 (2)	0.029 (2)	0.031 (2)	−0.0092 (17)	−0.0030 (17)	−0.0068 (18)
C25	0.113 (5)	0.034 (3)	0.022 (2)	−0.035 (3)	0.002 (3)	−0.008 (2)
C26A	0.029 (4)	0.040 (5)	0.048 (6)	−0.006 (4)	−0.003 (4)	−0.023 (5)
C27A	0.040 (6)	0.052 (7)	0.049 (8)	−0.008 (6)	−0.006 (6)	−0.030 (7)
C28A	0.060 (8)	0.033 (5)	0.028 (5)	−0.008 (5)	0.000 (5)	−0.015 (4)
C29A	0.036 (5)	0.038 (5)	0.031 (5)	0.001 (4)	0.001 (4)	−0.010 (4)
C30A	0.033 (4)	0.039 (5)	0.031 (5)	−0.007 (4)	0.003 (4)	−0.018 (4)
C26B	0.049 (5)	0.030 (4)	0.029 (4)	−0.006 (4)	−0.006 (4)	−0.010 (4)
C27B	0.057 (7)	0.035 (5)	0.041 (6)	−0.010 (5)	0.004 (5)	−0.023 (4)
C28B	0.040 (6)	0.049 (6)	0.031 (6)	−0.013 (5)	0.004 (5)	−0.025 (5)
C29B	0.042 (5)	0.044 (5)	0.026 (4)	−0.012 (4)	−0.002 (3)	−0.010 (4)
C30B	0.036 (4)	0.035 (5)	0.031 (4)	−0.004 (4)	−0.005 (3)	−0.011 (4)

### Geometric parameters (Å, °)

**Table:**

Au1—Cl1	2.3043 (10)	C16—C17	1.520 (7)
Au1—As1	2.3411 (4)	C16—H16A	0.9700
Au2—Cl2	2.3005 (10)	C16—H16B	0.9700
Au2—As2	2.3398 (5)	C17—C18	1.524 (7)
As1—C13	1.929 (4)	C17—H17A	0.9700
As1—C1	1.930 (4)	C17—H17B	0.9700
As1—C7	1.937 (4)	C18—H18A	0.9700
As2—C19	1.925 (4)	C18—H18B	0.9700
As2—C25	1.931 (5)	C19—C24	1.400 (6)
As2—C18	1.940 (4)	C19—C20	1.403 (6)
C1—C2	1.392 (6)	C20—C21	1.380 (7)
C1—C6	1.394 (6)	C20—H20A	0.9300
C2—C3	1.393 (6)	C21—C22	1.399 (7)
C2—H2A	0.9300	C21—H21A	0.9300
C3—C4	1.382 (8)	C22—C23	1.387 (7)
C3—H3A	0.9300	C22—H22A	0.9300
C4—C5	1.390 (7)	C23—C24	1.388 (7)
C4—H4A	0.9300	C23—H23A	0.9300
C5—C6	1.384 (7)	C24—H24A	0.9300
C5—H5A	0.9300	C25—C26A	1.240 (11)
C6—H6A	0.9300	C25—C26B	1.251 (11)
C7—C8	1.373 (6)	C25—C30B	1.537 (11)
C7—C12	1.400 (6)	C25—C30A	1.611 (12)
C8—C9	1.402 (6)	C26A—C27A	1.398 (16)
C8—H8A	0.9300	C26A—H26A	0.9300
C9—C10	1.389 (8)	C27A—C28A	1.351 (18)
C9—H9A	0.9300	C27A—H27A	0.9300
C10—C11	1.374 (8)	C28A—C29A	1.375 (16)
C10—H10A	0.9300	C28A—H28A	0.9300
C11—C12	1.401 (6)	C29A—C30A	1.404 (13)
C11—H11A	0.9300	C29A—H29A	0.9300
C12—H12A	0.9300	C30A—H30A	0.9300
C13—C14	1.524 (6)	C26B—C27B	1.403 (12)
C13—H13A	0.9700	C26B—H26B	0.9300
C13—H13B	0.9700	C27B—C28B	1.376 (17)
C14—C15	1.530 (6)	C27B—H27B	0.9300
C14—H14A	0.9700	C28B—C29B	1.383 (16)
C14—H14B	0.9700	C28B—H28B	0.9300
C15—C16	1.523 (6)	C29B—C30B	1.383 (13)
C15—H15A	0.9700	C29B—H29B	0.9300
C15—H15B	0.9700	C30B—H30B	0.9300
			
Cl1—Au1—As1	174.77 (3)	C15—C16—H16B	109.2
Cl2—Au2—As2	175.14 (3)	H16A—C16—H16B	107.9
C13—As1—C1	103.67 (19)	C16—C17—C18	114.4 (4)
C13—As1—C7	104.75 (18)	C16—C17—H17A	108.7
C1—As1—C7	105.48 (17)	C18—C17—H17A	108.7
C13—As1—Au1	115.15 (13)	C16—C17—H17B	108.7
C1—As1—Au1	114.54 (14)	C18—C17—H17B	108.7
C7—As1—Au1	112.21 (14)	H17A—C17—H17B	107.6
C19—As2—C25	102.5 (2)	C17—C18—As2	115.9 (3)
C19—As2—C18	107.7 (2)	C17—C18—H18A	108.3
C25—As2—C18	101.2 (3)	As2—C18—H18A	108.3
C19—As2—Au2	110.57 (14)	C17—C18—H18B	108.3
C25—As2—Au2	117.1 (2)	As2—C18—H18B	108.3
C18—As2—Au2	116.39 (14)	H18A—C18—H18B	107.4
C2—C1—C6	120.8 (4)	C24—C19—C20	119.4 (4)
C2—C1—As1	117.3 (3)	C24—C19—As2	118.0 (3)
C6—C1—As1	121.8 (3)	C20—C19—As2	122.6 (3)
C1—C2—C3	119.1 (4)	C21—C20—C19	119.7 (4)
C1—C2—H2A	120.5	C21—C20—H20A	120.1
C3—C2—H2A	120.5	C19—C20—H20A	120.1
C4—C3—C2	120.4 (4)	C20—C21—C22	120.5 (4)
C4—C3—H3A	119.8	C20—C21—H21A	119.7
C2—C3—H3A	119.8	C22—C21—H21A	119.7
C3—C4—C5	120.1 (5)	C23—C22—C21	120.1 (4)
C3—C4—H4A	120.0	C23—C22—H22A	120.0
C5—C4—H4A	120.0	C21—C22—H22A	120.0
C6—C5—C4	120.3 (5)	C22—C23—C24	119.7 (4)
C6—C5—H5A	119.8	C22—C23—H23A	120.2
C4—C5—H5A	119.8	C24—C23—H23A	120.2
C5—C6—C1	119.3 (4)	C23—C24—C19	120.6 (4)
C5—C6—H6A	120.3	C23—C24—H24A	119.7
C1—C6—H6A	120.3	C19—C24—H24A	119.7
C8—C7—C12	120.5 (4)	C26A—C25—C26B	59.0 (7)
C8—C7—As1	118.9 (3)	C26A—C25—C30B	66.8 (7)
C12—C7—As1	120.6 (3)	C26B—C25—C30B	117.9 (7)
C7—C8—C9	120.1 (4)	C26A—C25—C30A	113.6 (7)
C7—C8—H8A	120.0	C26B—C25—C30A	76.6 (7)
C9—C8—H8A	120.0	C30B—C25—C30A	101.0 (6)
C10—C9—C8	119.5 (5)	C26A—C25—As2	130.7 (7)
C10—C9—H9A	120.2	C26B—C25—As2	124.9 (6)
C8—C9—H9A	120.2	C30B—C25—As2	112.5 (5)
C11—C10—C9	120.6 (4)	C30A—C25—As2	114.6 (5)
C11—C10—H10A	119.7	C25—C26A—C27A	127.1 (10)
C9—C10—H10A	119.7	C25—C26A—H26A	116.5
C10—C11—C12	120.2 (5)	C27A—C26A—H26A	116.5
C10—C11—H11A	119.9	C28A—C27A—C26A	120.3 (10)
C12—C11—H11A	119.9	C28A—C27A—H27A	119.9
C7—C12—C11	119.1 (4)	C26A—C27A—H27A	119.9
C7—C12—H12A	120.5	C27A—C28A—C29A	121.6 (9)
C11—C12—H12A	120.5	C27A—C28A—H28A	119.2
C14—C13—As1	110.6 (3)	C29A—C28A—H28A	119.2
C14—C13—H13A	109.5	C28A—C29A—C30A	118.7 (9)
As1—C13—H13A	109.5	C28A—C29A—H29A	120.6
C14—C13—H13B	109.5	C30A—C29A—H29A	120.6
As1—C13—H13B	109.5	C29A—C30A—C25	118.1 (8)
H13A—C13—H13B	108.1	C29A—C30A—H30A	121.0
C13—C14—C15	114.2 (4)	C25—C30A—H30A	121.0
C13—C14—H14A	108.7	C25—C26B—C27B	123.6 (9)
C15—C14—H14A	108.7	C25—C26B—H26B	118.2
C13—C14—H14B	108.7	C27B—C26B—H26B	118.2
C15—C14—H14B	108.7	C28B—C27B—C26B	117.6 (10)
H14A—C14—H14B	107.6	C28B—C27B—H27B	121.2
C16—C15—C14	115.4 (4)	C26B—C27B—H27B	121.2
C16—C15—H15A	108.4	C27B—C28B—C29B	122.0 (10)
C14—C15—H15A	108.4	C27B—C28B—H28B	119.0
C16—C15—H15B	108.4	C29B—C28B—H28B	119.0
C14—C15—H15B	108.4	C30B—C29B—C28B	119.7 (9)
H15A—C15—H15B	107.5	C30B—C29B—H29B	120.2
C17—C16—C15	111.8 (4)	C28B—C29B—H29B	120.2
C17—C16—H16A	109.2	C29B—C30B—C25	116.0 (8)
C15—C16—H16A	109.2	C29B—C30B—H30B	122.0
C17—C16—H16B	109.2	C25—C30B—H30B	122.0
			
C13—As1—C1—C2	−51.8 (4)	C24—C19—C20—C21	−1.1 (7)
C7—As1—C1—C2	−161.6 (4)	As2—C19—C20—C21	−178.0 (4)
Au1—As1—C1—C2	74.5 (4)	C19—C20—C21—C22	2.0 (7)
C13—As1—C1—C6	132.9 (5)	C20—C21—C22—C23	−1.4 (7)
C7—As1—C1—C6	23.0 (5)	C21—C22—C23—C24	0.1 (7)
Au1—As1—C1—C6	−100.9 (4)	C22—C23—C24—C19	0.8 (7)
C6—C1—C2—C3	0.1 (8)	C20—C19—C24—C23	−0.2 (7)
As1—C1—C2—C3	−175.2 (4)	As2—C19—C24—C23	176.8 (3)
C1—C2—C3—C4	0.5 (8)	C19—As2—C25—C26A	111.1 (9)
C2—C3—C4—C5	−1.5 (10)	C18—As2—C25—C26A	−137.7 (9)
C3—C4—C5—C6	1.9 (12)	Au2—As2—C25—C26A	−10.1 (9)
C4—C5—C6—C1	−1.2 (11)	C19—As2—C25—C26B	−172.1 (8)
C2—C1—C6—C5	0.3 (9)	C18—As2—C25—C26B	−60.9 (8)
As1—C1—C6—C5	175.4 (5)	Au2—As2—C25—C26B	66.7 (9)
C13—As1—C7—C8	128.8 (4)	C19—As2—C25—C30B	33.0 (6)
C1—As1—C7—C8	−122.1 (4)	C18—As2—C25—C30B	144.2 (5)
Au1—As1—C7—C8	3.2 (4)	Au2—As2—C25—C30B	−88.2 (5)
C13—As1—C7—C12	−51.0 (4)	C19—As2—C25—C30A	−81.7 (6)
C1—As1—C7—C12	58.0 (4)	C18—As2—C25—C30A	29.6 (6)
Au1—As1—C7—C12	−176.6 (3)	Au2—As2—C25—C30A	157.2 (5)
C12—C7—C8—C9	−0.5 (7)	C26B—C25—C26A—C27A	64.7 (13)
As1—C7—C8—C9	179.6 (4)	C30B—C25—C26A—C27A	−83.6 (14)
C7—C8—C9—C10	0.0 (7)	C30A—C25—C26A—C27A	8.7 (16)
C8—C9—C10—C11	0.5 (8)	As2—C25—C26A—C27A	176.0 (10)
C9—C10—C11—C12	−0.5 (7)	C25—C26A—C27A—C28A	−5(2)
C8—C7—C12—C11	0.5 (6)	C26A—C27A—C28A—C29A	0(2)
As1—C7—C12—C11	−179.6 (3)	C27A—C28A—C29A—C30A	−0.6 (18)
C10—C11—C12—C7	0.0 (7)	C28A—C29A—C30A—C25	4.8 (15)
C1—As1—C13—C14	−179.7 (3)	C26A—C25—C30A—C29A	−8.6 (13)
C7—As1—C13—C14	−69.3 (4)	C26B—C25—C30A—C29A	−55.6 (10)
Au1—As1—C13—C14	54.4 (3)	C30B—C25—C30A—C29A	60.7 (10)
As1—C13—C14—C15	168.3 (3)	As2—C25—C30A—C29A	−178.1 (7)
C13—C14—C15—C16	60.8 (7)	C26A—C25—C26B—C27B	−55.6 (11)
C14—C15—C16—C17	61.3 (6)	C30B—C25—C26B—C27B	−22.5 (14)
C15—C16—C17—C18	165.4 (4)	C30A—C25—C26B—C27B	73.0 (11)
C16—C17—C18—As2	61.0 (5)	As2—C25—C26B—C27B	−176.2 (8)
C19—As2—C18—C17	−80.8 (4)	C25—C26B—C27B—C28B	14.1 (17)
C25—As2—C18—C17	172.0 (4)	C26B—C27B—C28B—C29B	−1.1 (18)
Au2—As2—C18—C17	44.0 (4)	C27B—C28B—C29B—C30B	−0.9 (17)
C25—As2—C19—C24	−104.7 (4)	C28B—C29B—C30B—C25	−7.1 (13)
C18—As2—C19—C24	149.0 (3)	C26A—C25—C30B—C29B	49.4 (9)
Au2—As2—C19—C24	20.8 (4)	C26B—C25—C30B—C29B	18.8 (12)
C25—As2—C19—C20	72.2 (4)	C30A—C25—C30B—C29B	−61.8 (9)
C18—As2—C19—C20	−34.1 (4)	As2—C25—C30B—C29B	175.6 (6)
Au2—As2—C19—C20	−162.3 (3)		

### Hydrogen-bond geometry (Å, °)

**Table:**

*D*—H···*A*	*D*—H	H···*A*	*D*···*A*	*D*—H···*A*
C17—H17B···Cl2^i^	0.97	2.79	3.754 (5)	172
C18—H18A···Cl1^ii^	0.97	2.80	3.701 (5)	155

Symmetry codes: (i) −*x*+1, −*y*+1, −*z*; (ii) −*x*, −*y*+1, −*z*.
